# A low profile super UWB- MIMO antenna with d-shaped for satellite communications, 5G and beyond applications

**DOI:** 10.1038/s41598-025-96017-3

**Published:** 2025-05-05

**Authors:** Hesham A. Mohamed, Mohamed Aboualalaa

**Affiliations:** 1 Microstrip Department, Electronics Research Institute, Cairo, 11843 Egypt; 2 National Telecommunications Regulatory Authority, Giza, 12577 Egypt

**Keywords:** Fifth-generation (5G), Super ultra-wideband (S UWB) system, Multiple-input multiple-output (MIMO), Envelope correlation coefficient (ECC), Electrical and electronic engineering, Energy science and technology, Energy harvesting

## Abstract

This study presents a new compact single-layer microstrip 4-port super UWB MIMO antenna designed to operate in the frequency range of (2.5–50) GHz, achieving an impressive 320.2% impedance bandwidth. The antenna is based on a unique d-shaped geometry, specially tailored for applications in satellite communication, 5G, and Beyond, covering various bands including S-, C-, 4G LTE, sub-6 GHz, UWB, and X, ka-, k-, and ku- satellite communication bands. This aligns with Europe’s efforts to harmonize by designating the 26 GHz band as a pioneer band for 5G. Additionally, 5G millimeter-wave frequencies are increasingly used in Internet of Things (IoT) applications and industrial automation. In the 5G spectrum, the midband (FR1) spans 1 GHz to 7 GHz, while the high band (FR2) covers frequencies from 24 to 52 GHz and smart wearable devices, vehicle radars, satellite communications, and smart 5G remote sensors devices. The antenna system comprises four orthogonally symmetrically placed identical radiating elements, each featuring a d-shaped patch. The metallic ground plane incorporates a curvature with a simple half-circle shape, effectively enhancing the antenna’s matched bandwidth by altering the current distribution, consequently affecting the inductance and capacitance within the ground plane. A MIMO structure comprising four elements of the proposed antenna is introduced. Both simulation and experiments of the MIMO system demonstrate the antenna’s impressive performance, showcasing an impedance bandwidth of 2.5 to 50 GHz, a whole-working bandwidth isolation exceeding 20 dB, an envelope correlation coefficient (ECC) below 0.013, and a significant increase in diversity gain (DG) of over 9.98 dBi. The antenna exhibits excellent radiation characteristics and a stable gain, making it highly suitable for UWB MIMO system applications.

## Introduction

Lately, within the realm of wireless communication systems, there has been a development of antenna configurations catering not only to ultra-wideband (UWB) uses (ranging from 3.10 GHz to 10.6 GHz)^1,2^, but also to Super UWB (S-UWB) antennas exhibiting bandwidth ratios exceeding 10 to 1. These advancements have introduced fresh avenues for diverse applications, reshaping wireless technologies across domains like microwave imaging, cognitive radio, sensing networks, high-speed wireless data transmission, X-band Radar, and K-band Satellite systems^3–6^. The growing quantity and improved caliber of applications necessitate the use of ultra-wideband (UWB) antennas capable of satisfying the demands for higher data rates^7^.

Regarding potential applications of the conceivable MIMO antenna, the current emphasis in scholarly works has predominantly revolved around the design aspect. Despite this, prevalent technologies like 4G, WiFi, and WLAN still rely on microwave sub-6 GHz and mm-wave frequency bands^8–16^. This is consistent with European Harmonization’s adoption^17^ of the 26 GHz frequency as the European 5G band. Additionally, 5G millimeter-wave frequencies are used in industrial automation and Internet of Things (IoT) applications. Midband (FR1) 1 GHz-7 GHz and high band/mmWave (FR2) with band 24 GHz to 52 GHz are similar to 5G frequencies.

This continued utilization is attributed to the complexities associated with creating radio frequency front ends capable of functioning within the millimeter-wave spectrum, where signal attenuation becomes a challenge. In response to the expanding user demands, the fifth generation (5G) of mobile communication technology strives to furnish swift data rates measured in gigabits per second, accompanied by minimal latency.

Moreover, UWB technology operates through abbreviated bursts of electromagnetic signals. Enhancing communication quality and channel capacity presents a substantial challenge, demanding compact, wide bandwidth, and high gain antennas. The emergence of data-centric applications in mobile wireless technology has paved the way for integrating multiple-input multiple-output (MIMO) functionality into smartphones. This integration leverages multi-path transmission and reception to amplify data throughput through various channels^18–30^. MIMO antennas have emerged as a valuable remedy to address the aforementioned concern. These intelligent electronic devices possess diverse capabilities like sensing, remote accessibility, automation, health monitoring, and more. To fully exploit these features, seamless wireless communication with ample bandwidth is essential^3^. Herein, ultrawideband (UWB) technology steps in to meet the escalating bandwidth demand. Operating within the unlicensed 3.1 to 10.6 GHz frequency range, UWB is tailored for commercial applications^1^. The integration of the expansive 7.5 GHz bandwidth into the UWB system has garnered substantial attention, driving noteworthy progress in wireless communication systems. The design of the UWB antenna holds significant importance within the UWB system, as it must adhere to specific temporal and frequency domain attributes across the impedance bandwidth. Monopole antennas offer substantial potential for UWB wireless communication due to their favorable attributes such as compact size, extensive bandwidth, and cost-effectiveness^3,4^.

The study of this paper motivates to a compact size 4-port super UWB MIMO antenna designed to increase with the working frequency of covering super UWB range of 2.5 –50 GHz, to be operate at UWB and lower with upper 5G bands, offering wideband capability that provides greater flexibility and versatility for high data rate applications. 3D electromagnetic software (Computer Simulation Technology (CST)) is utilized to design and simulate the proposed antenna design. The novelty of the article and further improvement as design super UWB-MIMO antenna system to cover lower and higher frequencies applications. We study on characteristic mode analysis (CMA) to identify the modes responsible for this large bandwidth. In the 2-element MIMO system we are demonstrating a wide apparent bandwidth and an isolation in both cases. 90-degree and 0 degree in the 90-degree or orthogonal arrangement of elements exhibits a higher level of isolation compared to the 0-degree arrangement of elements along the vertical axis. We provided a common ground with narrow rectangular strip integrated MIC in protruded from the ground plane located between the four antennas and decoupling element, this technique achieves high isolation between antennas. In order to save space and increase the isolation between the elements, the suggested antenna elements are arranged perpendicularly to handle mission-critical positioning the UWB technology is developed specifically to meet above requirement with an overall size of 70 × 70 × 1.575 mm.

The structure of this study is outlined as follows: firstly, the evolution of the design and the parametric analysis of the individual antenna are detailed, including a juxtaposition of the simulated outcomes against the experimental findings. Moving on to secondly, the subsequent part centers on the design of the MIMO antenna and its assessment, encompassing measurements of reflection coefficients, gain, and radiation pattern. Thirdly, show the scenarios of the recommended 4-port MIMO antenna are encapsulates the final insights drawn from this research. Finally, provides comparisons between simulations evaluated by CST from the proposed design and measurements obtained by collected the Predicted results. This work is concluded with references in Sect. 6. Lastly, in section seven, acknowledgments are extended.

## Methods and design construction

### Super UWB single-element antenna design

**Fig. 1:**
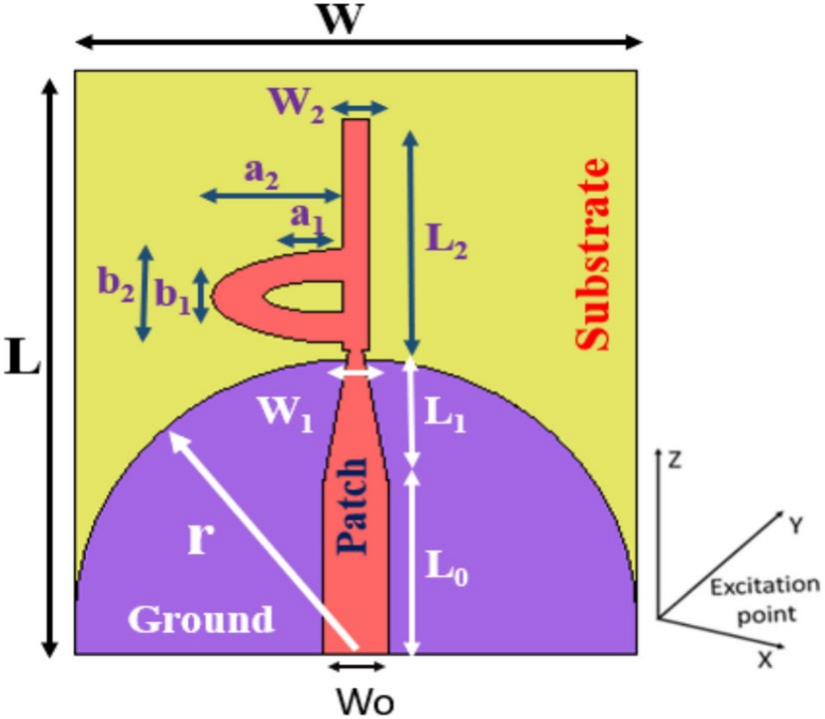
2D Geometry of the proposed antenna.

The proposed antenna is a flat printed design with compact overall dimensions measuring 32 × 31 × 1.575 mm2. Figure 1 illustrates the 2D layout of the monopole antenna design. The antenna features a simplified configuration, comprising a patch resembling the letter "d" and a semicircular ground plane with a rectangular truncation at the base. The chosen substrate for the antenna is Roger RT/Duroid 3003, possessing a thickness of h = 1.575 mm, a loss tangent of 0.0012, and a relative permittivity of 3. The Rogers 3003 substrate has the advantages of high conductivity, superior relability, and mechanical stabilities designers working in the field of high-frequency. The antenna’s dimensions were meticulously selected and fine-tuned using Microwave Studio 2022 CST ver. 22. This software was employed to optimize the design parameters to achieve optimal performance metrics. The proposed antenna design underwent a series of steps to arrive at the final version, as outlined in the subsequent section. The dimensions indicated in Fig. 1 are tabulated in Table 1. The antenna is fed through a 50 Ω feeding line, characterized by a width of Wo = 3.8 mm and a length of Lo = 10.5 mm.

**Table 1:** single antenna geometry described (all dimensions in mm).

*W*	*L*	*W*_*o*_	*L*_*o*_	*h*	*W*_*1*_	*Wg*
31	33	3.8	10.5	1.575	0.8	70
*L*_*1*_	*L*_*2*_	*r*	*D*_*1*_	*D*_*2*_	tan σ	*Lg*
8	14.5	16	11	7.4	0.0026	70
a1	a2	b1	b2	*g0*	*Ls*	*Ws*
4.4	7.5	5.7	2	0.5	14	1

### Design procedure of the super UWB single Antenna

This section presents and discusses the step-by-step process involved in crafting the developed antenna. The comprehensive layout of the UWB antenna is depicted in Fig. 1. Notably, the antenna comprises a radiating patch formed through impedance matching of the 50 Ω feedline (Wo, Lo), alongside two distinct central circular patches featuring inner incisions to achieve the desired frequency band-shape slots on the upper surface. Additionally, a semicircular ground plane with a rectangular truncation at the base contributes to the structure. The ground plane is curved and modified that change the current distribution and affect the inductance and capacitance, to exhibit wide the impedance bandwidth. The UWB antenna under consideration is powered using a 50 Ω microstrip line. For the design of the proposed antenna, the substrate employed is RT/Duroid 3003 with a thickness of 1.575 mm, a relative permittivity of 3, and a loss tangent of 0.0025.

The incorporation of curvature in the design influences current distribution, affecting inductance and capacitance properties in the ground plane. These modifications contribute to achieving a broad bandwidth for the antenna. A key advantage of our design lies in its straight forwardness and planar structure, rendering it easily compatible with other microwave circuits. The specific design dimensions are listed in Table 1.

**Fig. 2:**
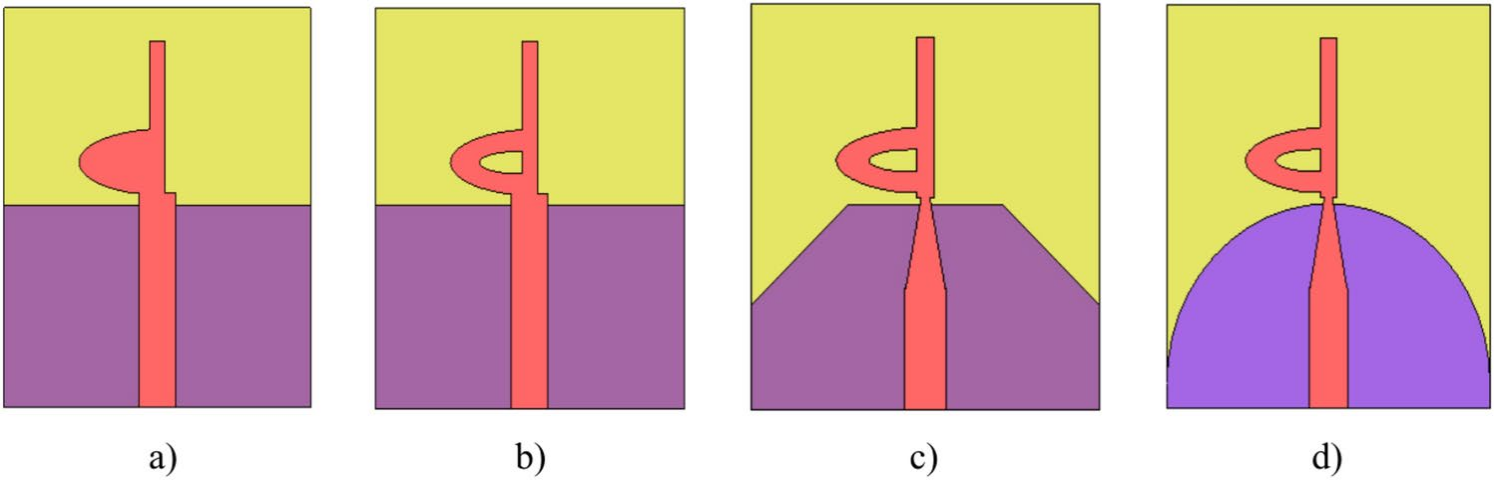
(**a**–**d**) Steps taken to evaluate the design procedure of the antenna.

**Fig. 3:**
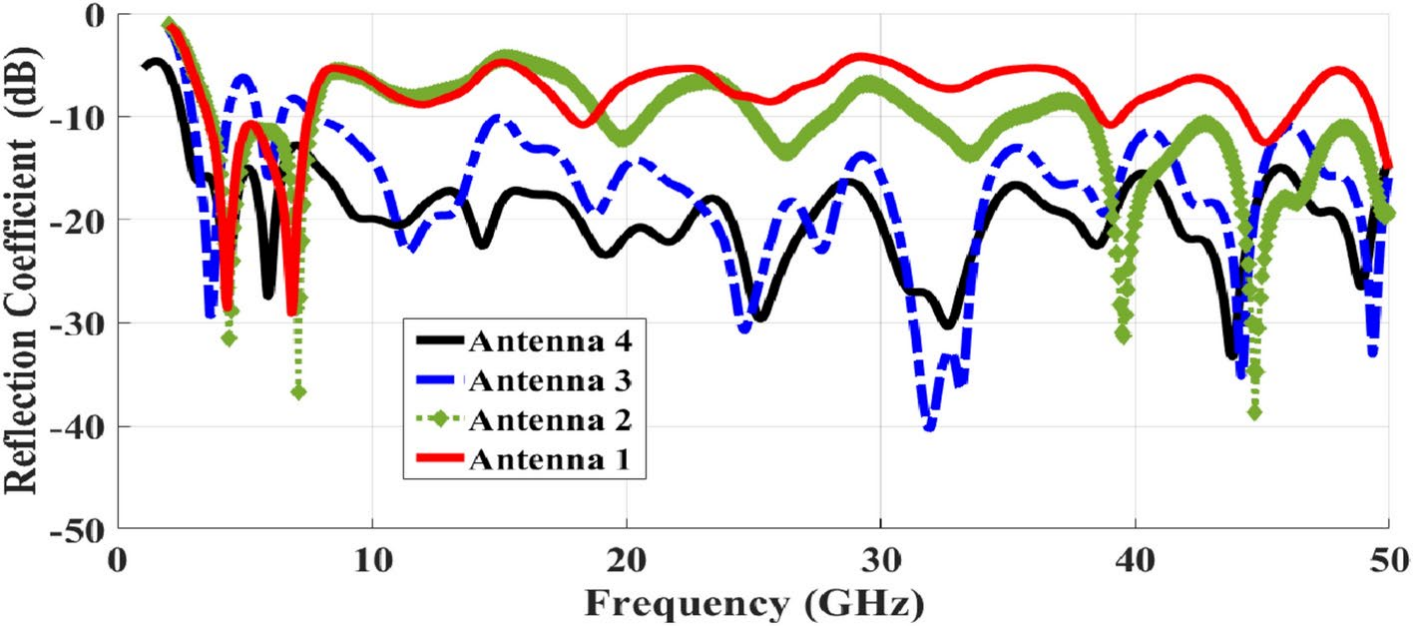
Simulated S11 results for several UWB single antenna design steps.

A sleeve antenna generally provides a wider bandwidth compared to a conventional monopole or dipole antenna. The sleeve structure acts as an impedance-matching element, reducing reflections and improving bandwidth performance. Therefore, we designed a patch monopole antenna based on an analogy to the coaxial sleeve antenna. We then modified the design to achieve better performance through trials with different shapes. The half-elliptical shape provided the best results. To further enhance the bandwidth, a tapered feeder was utilized, and the ground was optimized using partial, chamfer-shaped, and finally half-circle structures to achieve the desired UWB frequency range. The procedural steps for designing and optimizing the S_11_ responses of the Super UWB antenna are illustrated in Fig. 2. The S_11_ results are shown in Fig. 3.

To simulate the final proposed single antenna, CST 2022 software tools were employed. Figure 2 illustrates the successive design phases undertaken to arrive at the ultimate structure, while Fig. 3 presents the simulated return loss |S11| across various phases of the suggested antenna’s design procedure, encompassing different ground alterations and varying d shapes. These adjustments lead to a significant shift downwards in both the high-frequency resonant and low-frequency resonant modes. The alterations in physical length, though relatively minor, exert more pronounced effects on higher frequencies.

Figure 2(a) illustrates the initial iteration, presenting the basic layout of the developed antenna with a d-shaped patch and a partially implemented ground plane (Lo + L1 = 18.5 mm). Subsequently, in Fig. 2(b), the antenna exhibits unsatisfactory matching and extends bandwidth over the frequency range. The third phase, depicted in Fig. 2(c), is similar to the second phase but involves the addition of small sloped edges. This modification significantly impacts the impedance bandwidth.

As observed in Fig. 2(c), Antenna 3 demonstrates an impedance bandwidth spanning from 2.5 GHz to 50 GHz, featuring notches at 5.8 GHz and 8 GHz (indicated by the dashed blue curve). The ultimate design stage involves enhancing the developed antenna’s structure by introducing curvature and incisions into the metallic ground plane. This action elongates the electrical length through the addition of a semi-circular flawed ground structure in the ground plane. Consequently, the impedance bandwidth of the final configuration is notably enhanced, extending from 2.5 GHz to 50 GHz (depicted by the solid black curve). This adaptation ensures coverage of the entire UWB spectrum, as illustrated in stage 4 of Fig. 2(d).

**Fig. 4:**
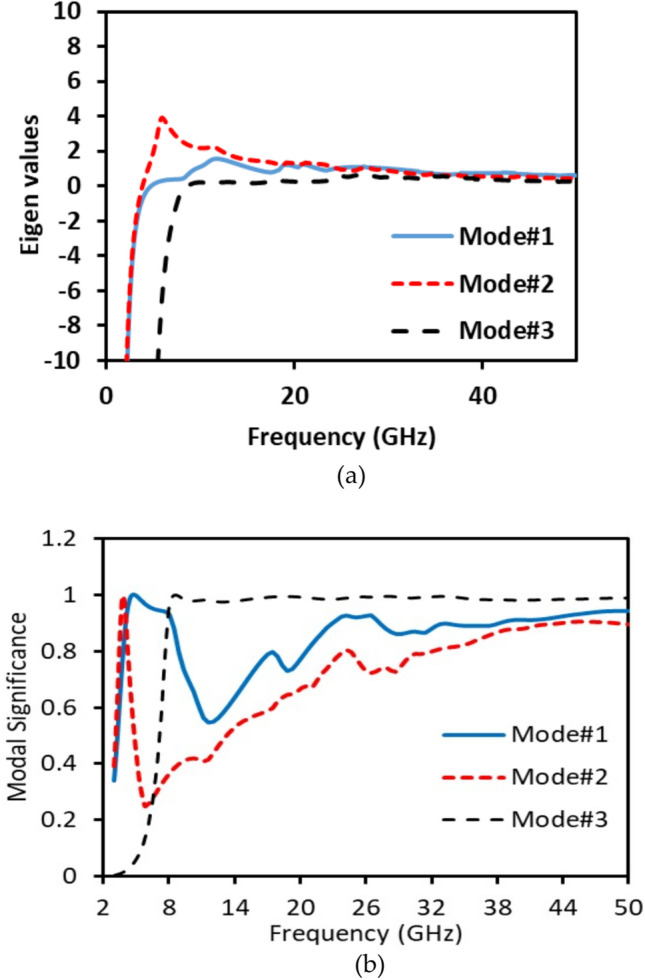
Simulated of modal significance of the proposed antenna design.

The characteristic mode analysis (CMA) is used to identify the modes of the proposed structure, with its dimensions optimized to radiate from 2.5 GHz to 50 GHz. Three modes are analyzed, as illustrated in Fig. 4. The CMA results indicate that three modes propagate simultaneously at the desired frequency bands, mode 1 dominates at the lower frequency and mode 3 dominates at the higher frequency band, as evident from the eigenvalue and modal significance results shown in Fig. 4 (a), and (b). The eigenvalue, representing the ratio of reactive to radiated power is zero, indicating the imaginary parts are zero at these resonance frequencies. Modal significance reflects each mode’s contribution to the overall electromagnetic response under an external source. Modes with a modal significance greater than 1/√2 are considered significant, while modes with a lower value are not. Therefore, at lower frequency band the most significant mode is mode 1, and at higher frequency band, mode 3 is the most significant mode.

### Current Distribution of UWB Antenna

**Fig. 5:**
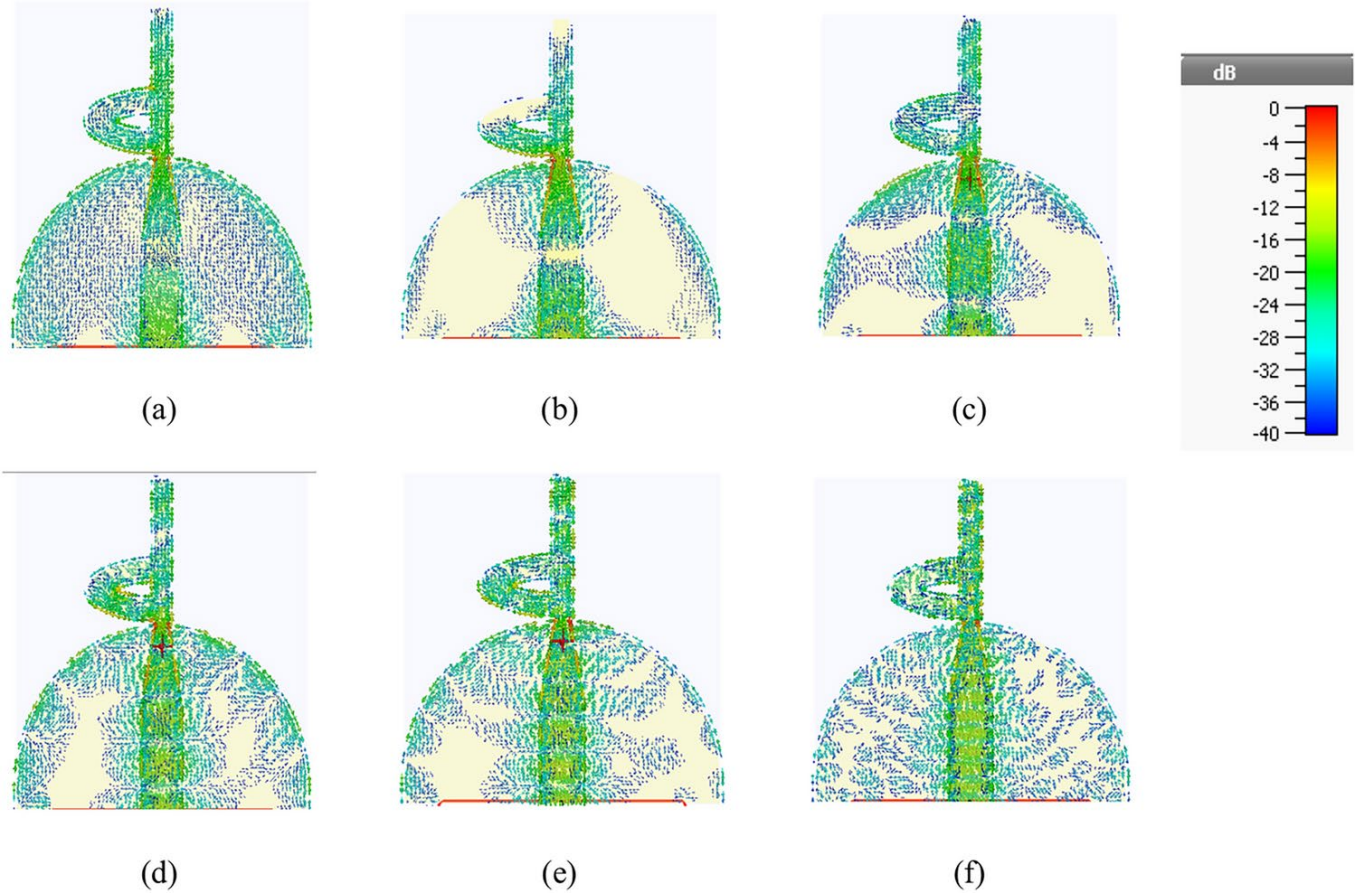
(**a**–**f**) Current distribution of the antenna at different frequencies; 3.1Hz, 6 GHz, 15 GHz, 25.5 GHz, 38 GHz and 48 GHz, respectively.

The simulated distribution of surface currents for the proposed antenna is presented in Fig. 5. This illustration highlights the coupling interaction between the radiating element and the geometric layout of the ground plane through the current distribution on the antenna. The current patterns are simulated at the first, second, and third resonance frequencies: 3.1 GHz, 6 GHz, 15 GHz, 25.5 GHz, 38 GHz, and 48 GHz. The distribution of current patterns reveals that a significant portion of the currents concentrates along the periphery of both the radiating patch and the ground plane. Conversely, the currents in the central areas of the patch and ground plane exhibit minimal strength. Furthermore, the current is observed to couple from the upper and lower edges of the ground plane to the patch via the microstrip feed line, subsequently radiating into the surrounding space.

The antenna’s size is reduced through the introduction of slots in the patch to facilitate radiation and by modifying the ground plane’s shape. These slots alter the way current flows and impact both inductance and capacitance, resulting in a broader bandwidth for the antenna. The strength of our design lies in its uncomplicated nature and flat configuration. The last version of the antenna is subjected to simulation using CST software. The specific dimensions of the design can be found in Table 1.

The modification applied to the upper edge of the partial ground plane successfully mitigates impedance variations in the antenna. This is achieved by redirecting the current path and generating a balanced current distribution across a smaller ground plane. As a result, the influence of the ground plane on the antenna’s performance is diminished. Nonetheless, at higher frequencies, the currents predominantly concentrate along the microstrip line and the intersection point between the patch and the ground plane. This leads to heightened currents on the ground plane compared to lower frequencies, subsequently causing a deterioration in impedance matching for modes reliant on traveling waves.

## Results

### Measured results of proposed super UWB Single Antenna

**Fig. 6:**
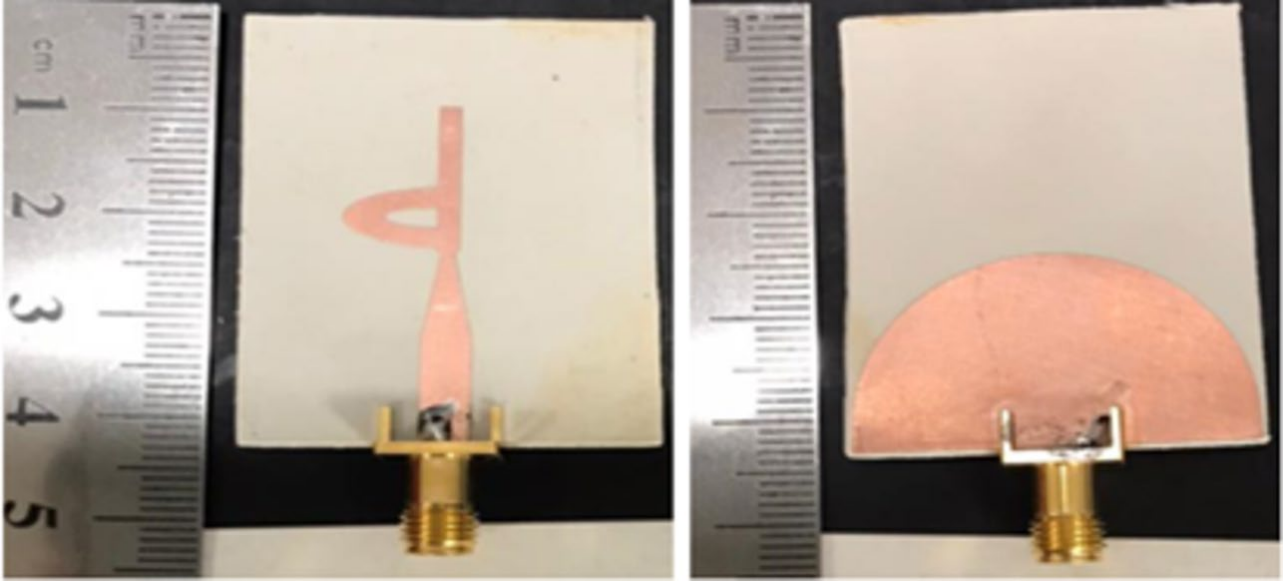
Manufactured prototype of the developed UWB antenna (**a**) front part and (**b**) back part.

**Fig. 7:**
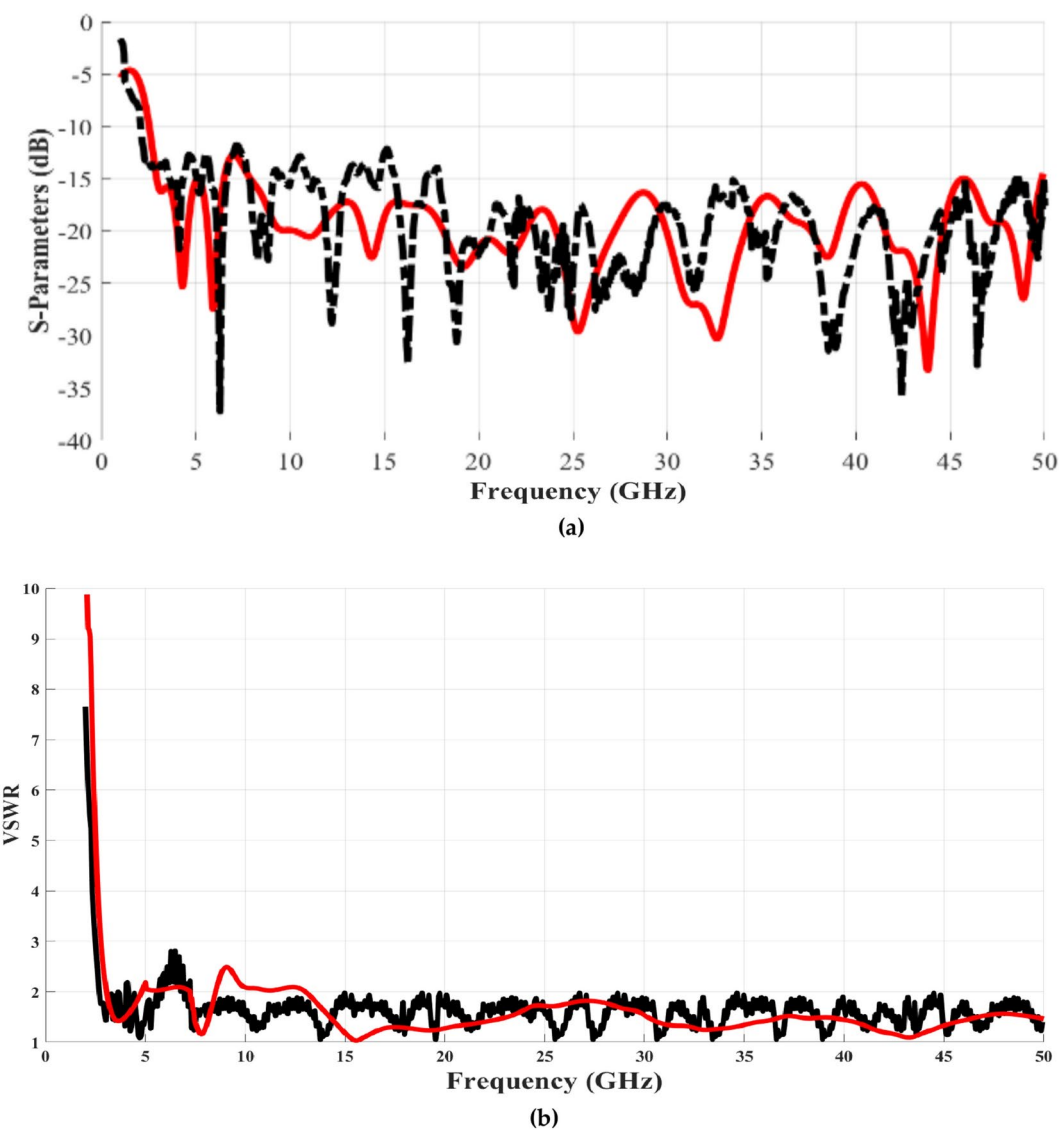
**(a)** The return loss Simulated and measured of the proposed antenna of the UWB antenna structure and (**b**) VSWR.

The proposed monopole antenna has been successfully manufactured and a prototype has been created, as depicted in Fig. 6 (a) for the front view and (b) for the rear view. Additionally, key antenna parameters such as the reflection coefficient |S11| and the voltage standing wave ratio (VSWR) at resonant frequencies have been evaluated. The reflection coefficients |S11|, demonstrating the optimal design of the proposed antenna, are presented in Fig. 7(a). These measurements were conducted utilizing a Rohde & Schwarz ZVA 67 Vector Network Analyzer, employing 50 Ω ports for both simulation and the UWB proposed antenna.

Figure 7(a) illustrates a comparison between the simulated and measured results for the reflection coefficient, showcasing a strong alignment between the two sets of data. This agreement verifies that the antenna effectively spans the entire spectrum required for various mobile and wireless applications. By defining the characteristic bandwidth impedance with a -10 dB reference to the reflection coefficient, the frequency ranges from 2.5 GHz up to 50 GHz has been identified as yielding super ultra-wideband performance. Furthermore, the VSWR, both simulated and measured, at different operational frequencies can be gleaned from the VSWR graph depicted in Fig. 7(b). On the whole, it is evident that there is a high degree of concordance between the simulated and measured values, affirming effective impedance matching throughout the frequency span extending from 2.5 to 50 GHz.

The disparities observed between the simulated and measured outcomes primarily stem from factors such as the impact of the measurement’s feeding cable. This is influenced by elements like the presence of the SMA connector, soldering joints employed to attach the SMA connector to the feeding line, and the influence of electromagnetic interference signals present in the surrounding environment. Additionally, the utilization of an idealized model during simulation, along with potential fabrication and measurement tolerances in the proposed antenna, contribute to the observed differences.

## MIMO antenna

### Two-Port MIMO Antenna configurations

**Fig. 8:**
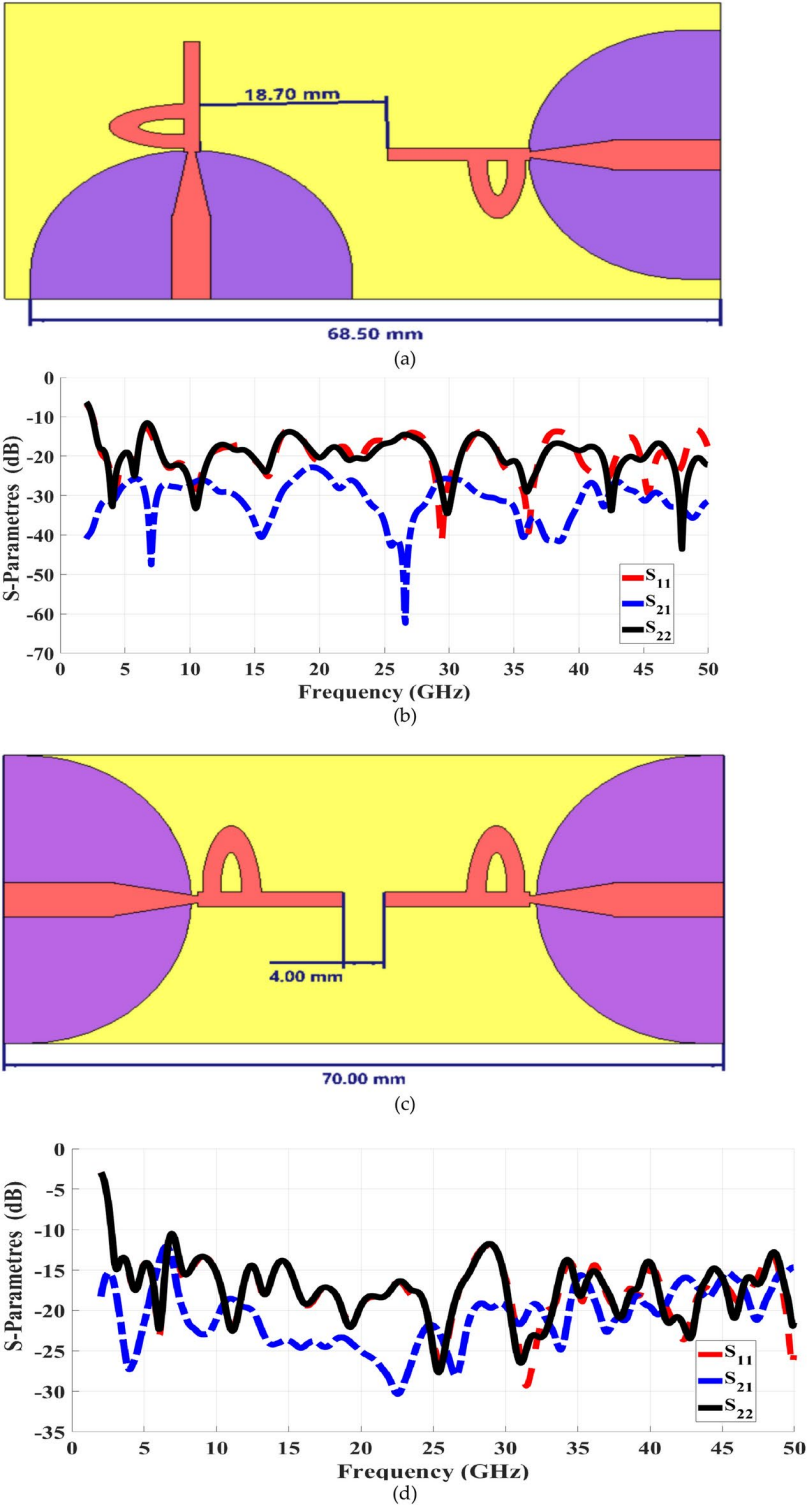
Two-port MIMO antenna, (**a**) 90 degree top/Botton view, (**b**) 90 degree S-parameters, (**c**)0 degree Design, and (**d**) 0 degree S-parameters.

The MIMO antenna is positioned at right angles to one another. Building upon the concept of the proposed UWB antenna, a configuration of two-port diversity arrays is devised to achieve effective signal reception within a multipath setting. This entails constructing a two-port Multiple-Input Multiple-Output (MIMO) antenna system by utilizing two instances of the proposed Super UWB patch. These elements are positioned 4 mm apart and are fed through a transmission line, as depicted in Fig. 8. This illustration displays the positioning of two 68 × 36 mm2 elements on the rogers 3003 substrate. The ground is first made of solid without any slots to get isolation of -18 dB. Then, a slot is added in the ground to act as decoupling structure to improve the isolation between the two elements up to -24 dB, which is reasonable for such antenna system.

To convert the single port to the four ports system, two configurations are considered: side-by-side and orthogonal orientations, both maintaining an equal edge-to-edge distance of 4 mm between elements, as shown in Fig. 8. The orthogonal orientation demonstrates superior performance compared to the side-by-side configuration. It enhances mutual coupling more effectively without the need for additional structures. Therefore, the orthogonal orientation is employed in the design of the four-port antenna.

Moreover, owing to the increasing influence of mutual coupling, the isolation curve surpasses the threshold of -15 dB at specific points within the designated operational frequency range. Additionally, the outcomes of the reverse reflection coefficient (S22) exhibit a mismatch with the results of the reflection coefficient (S11).

The MIMO diversity capabilities of the suggested antenna are assessed. Within MIMO systems, the extent of interaction between antenna elements is quantified by the envelope correlation coefficient (ECC). The diversity gain (DG) is linked to the ECC and characterizes the signal-to-interference ratio.

Figure 8 shows the reflection coefficient of the 2-element MIMO system with a separate ground, demonstrating a wide apparent bandwidth and an isolation in both cases. In Case 1,90-degree the antenna resonates within the frequency range of 2.5–50 GHz, the isolation greater than -20 dB. While in Case 2, 0-degree it resonates in all ranges, and within 35–50 GHz the isolation around -20 dB. The 90-degree or orthogonal arrangement of elements exhibits a higher level of isolation compared to the 0-degree arrangement of elements along the vertical axis.

### Four-Port MIMO Antenna design process

**Fig. 9:**
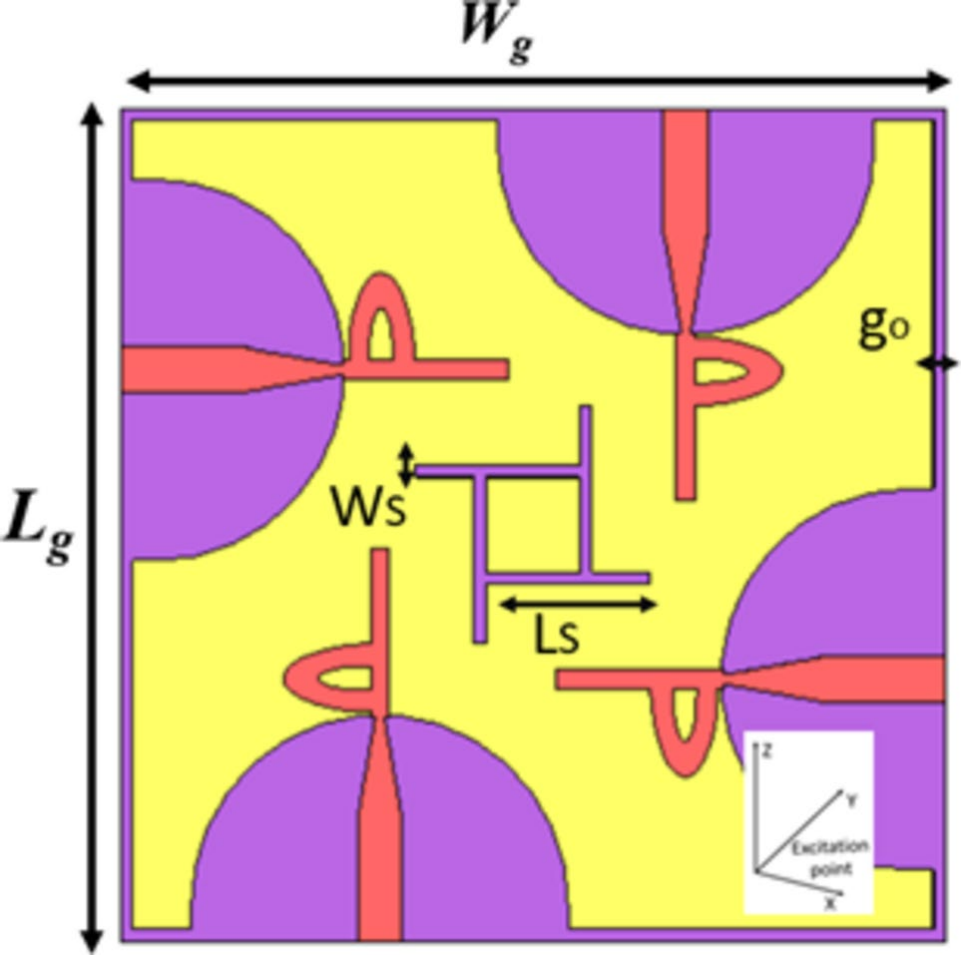
Full Structure view for proposed Four Port MIMO antenna system.

In order to facilitate MIMO functionality within the domains of 5G and sub-6 GHz wireless sensor networks, a multi-antenna architecture has been employed. This architecture incorporates spatial, polarization, and pattern diversity to ensure dependable communication. For effective operation within the Super ultrawide band, a four-port MIMO antenna system has been devised. The comprehensive configuration of this system is illustrated in Fig. 9. The design of the proposed MIMO antenna system involves the construction of four elements, each designed on a Rogers (RO3003) substrate with a relative permittivity of 3 and a loss tangent of 0.002. The substrate itself spans dimensions of 70 × 70 mm2. Given that the antenna elements are situated orthogonally to one another, the isolation between each port naturally exceeds 25 dB. We introduced a common ground connected between the MIMO elements featuring a simple, narrow rectangular strip protruding from the ground plane, positioned between the four antennas and the decoupling element (Ws and Ls). This technique achieves high isolation between the antennas, as illustrated in Fig. 9.

### Surface current distribution for four-port MIMO Antenna

**Fig. 10:**
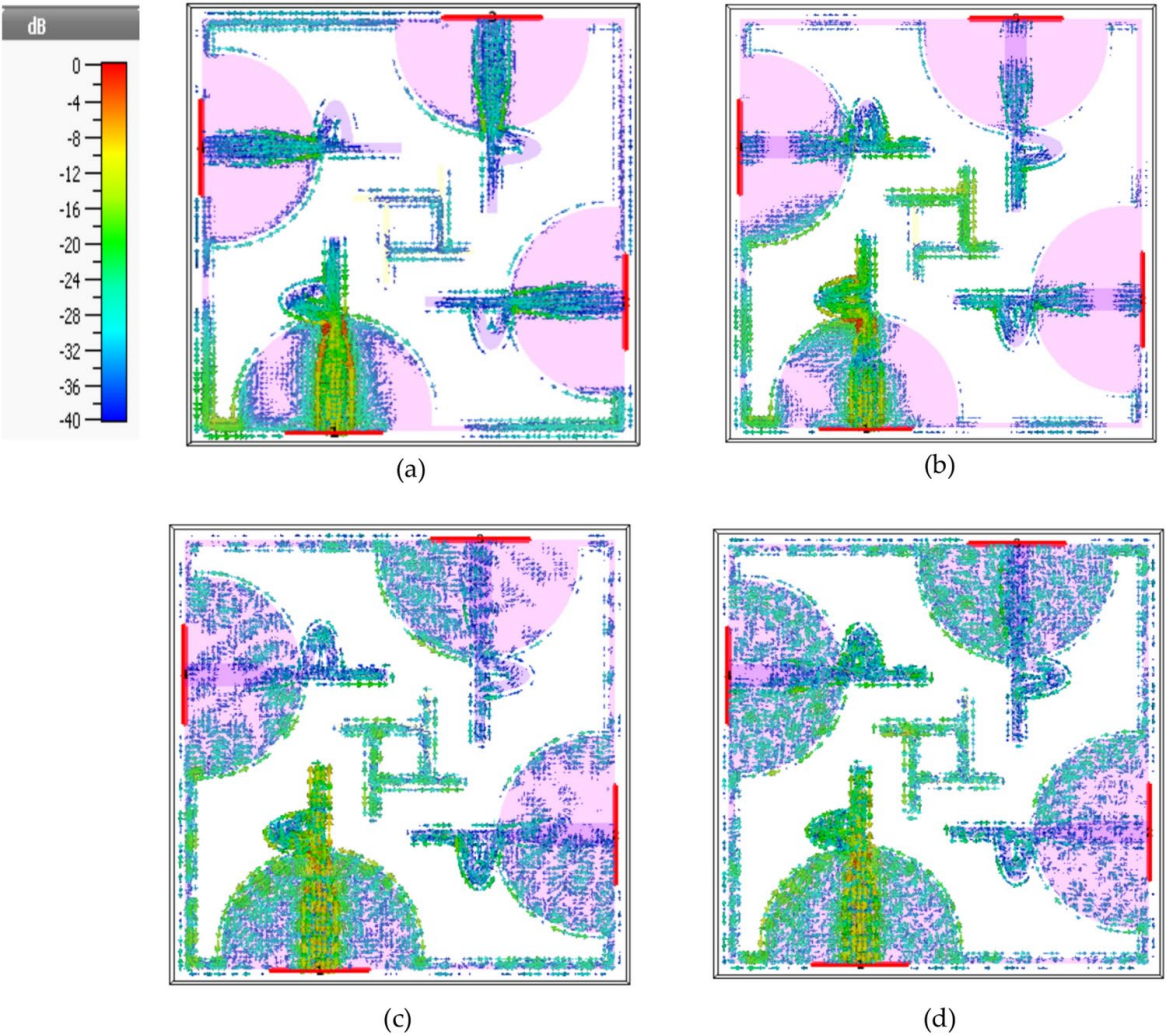
Surface current distribution results for Port # 1 excitation of UWB MIMO antenna structure at, (**a**) at 5.7 GHz, (**b**) at 18 GHz, (**c**) at 28 GHz, and (**d**) at 44 GHz.

The examination and assessment of surface current intensity across the radiator’s surface serve to validate its efficacy. Figures 10 a–d visually present the distribution of current density across the proposed structure. This analysis focuses particularly on resonance frequencies, showcasing the additional consideration of surface current distribution to understand the resonant behavior of the designed MIMO ultra-wideband antenna, as depicted in Fig. 10. This graphical representation elucidates how the current distribution of one component impacts the MIMO configuration of another element. The highest current density is observed at newly introduced patch edges, around the ground plane, stub conductor, and line feeder. This distribution pattern is particularly evident at the low resonances of 5.7 GHz, indicative of the first harmonic mode. For the middle and high resonances at 18, 28, and 44 GHz, the current distribution appears to embody higher frequencies. In crafting a UWB antenna for an extensive communication range, the surface current of the MIMO planar antenna plays a crucial role. It sustains harmonic order flux within the UWB system. The augmentation of surface current length is achieved through the incorporation of geometrically designed d-shaped radiator and the semi-circle shape integrated into the grounding component. Consequently, this approach facilitates the attainment of wideband operational attributes for the novel MIMO ultra-wideband antenna.

### Four-port MIMO scattering parameters

**Fig. 11:**
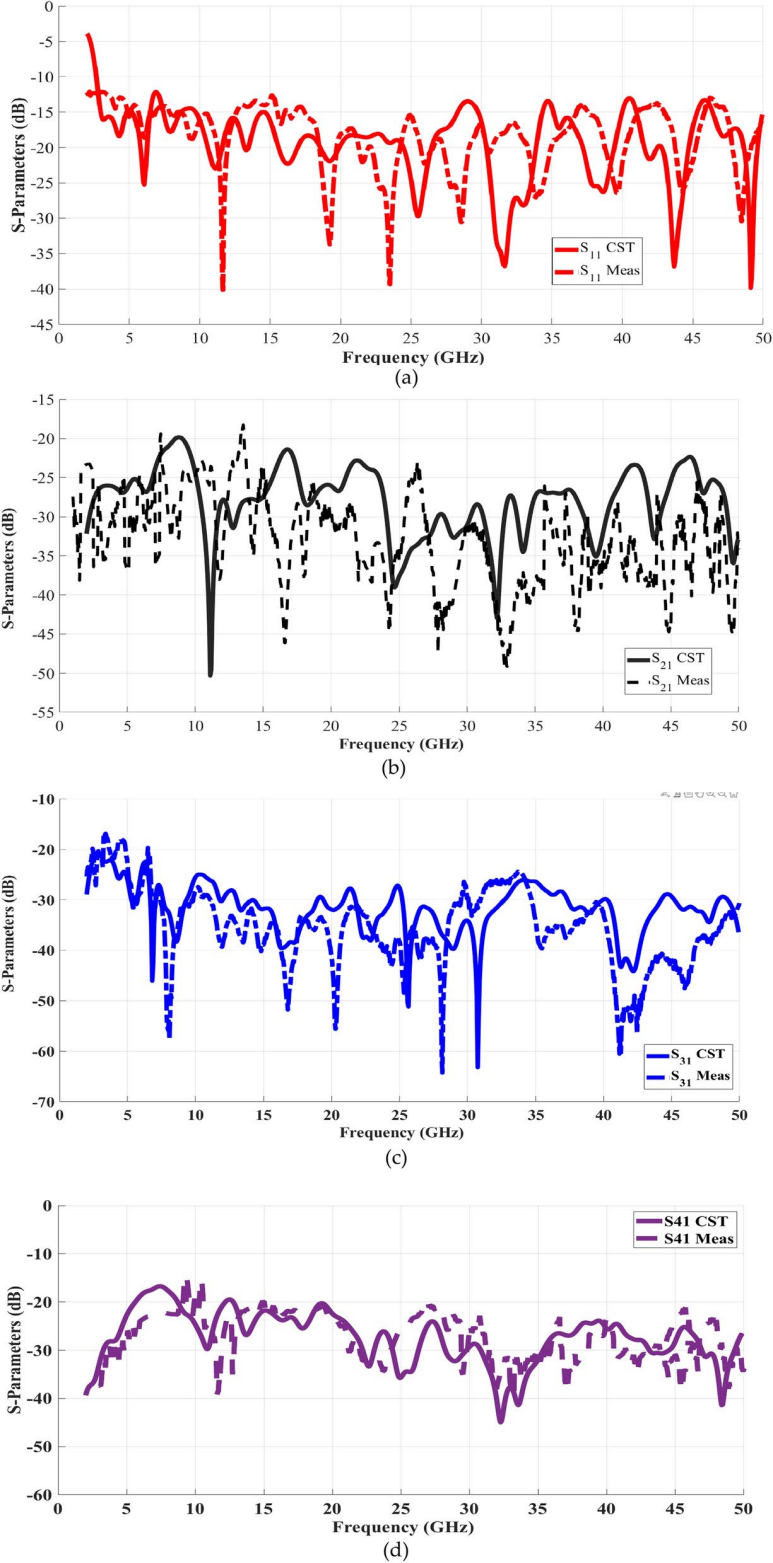
Measured and simulated S-Parameters of the proposed MIMO antenna.

This section illustrates the performance of the designed antenna through its S-Parameters. Figure 11a up to 11d present a comparison between the prototype-tested and CST-simulated results for the scattering parameters, specifically S11, S21, S31, and S41. The subsequent figure effectively demonstrates this comparison. The proposed antenna design successfully covers an extensive frequency range, spanning from 2.5 to 50 GHz, which exhibits noticeable conformity with the simulated outcomes. It’s worth noting that the antenna displays commendable isolation characteristics, with values oscillating between -22.5 and -19 dB. A marginal disparity between the simulated and measured results is observed. This difference predominantly stems from inherent flaws in the fabrication process and the configuration of measurement setups. Nonetheless, a marginal variation is attributed to factors like SMA connection losses, fabrication tolerances, dielectric properties, and conducting losses. Despite this minor difference, the antenna remains highly suitable for applications such as 5G mmWave, sub-6 GHz, and satellite communications.

**Fig. 12:**
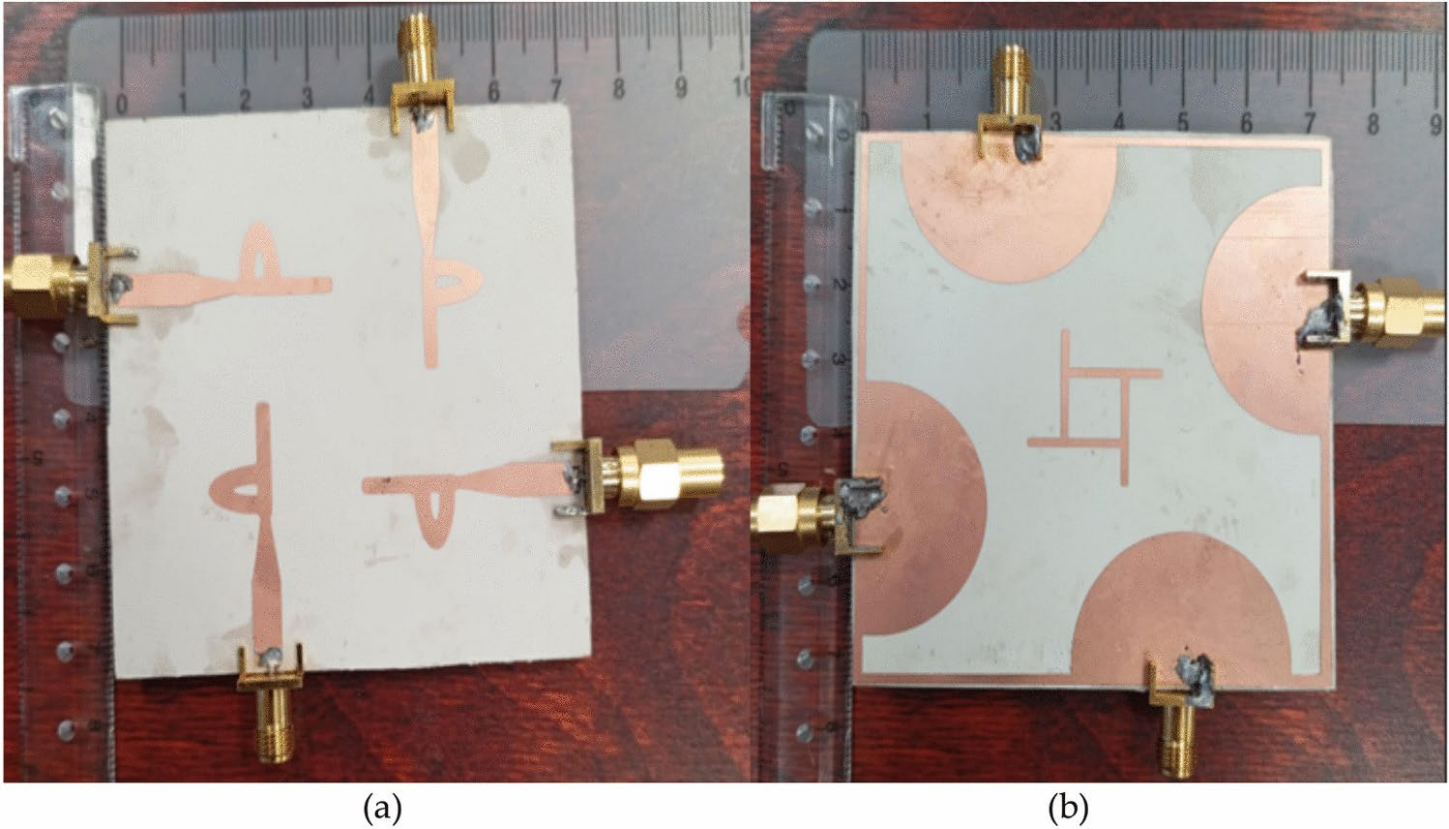
Fabricated MIMO antenna system (**a**) Top Patch (**b**) Bottom ground.

**Fig. 13:**
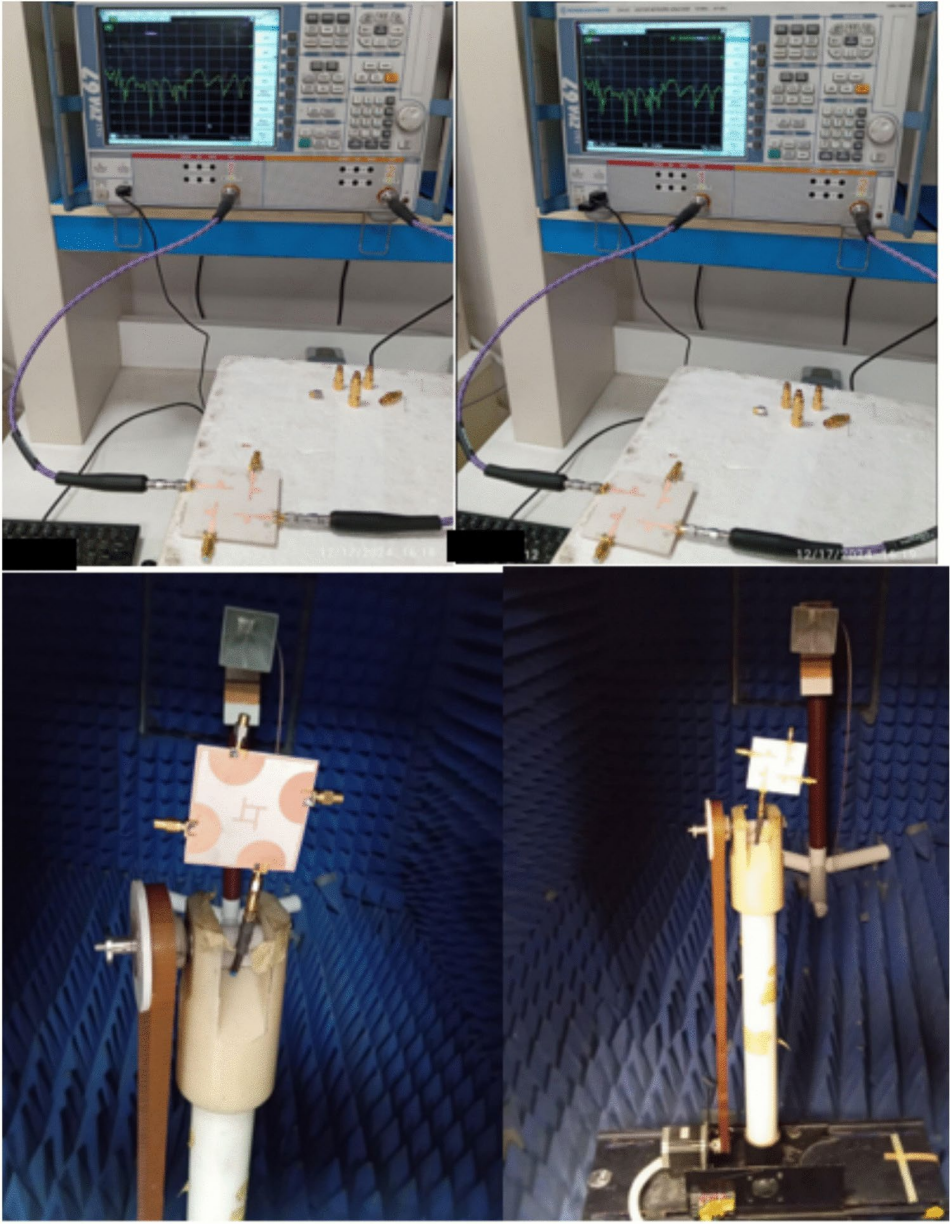
S-Parameters and Far-field measurement setup for radiation pattern and peak gain.

Additionally, Fig. 12 provides a depiction of the fabricated prototype of the suggested MIMO antenna undergoing radiation testing within an anechoic chamber as in Fig. 13.

### Far-field results

This section focuses on achieving radiation patterns and gain at certain frequencies where matching occurs. An image depicting the setup for measuring radiation patterns is presented in Fig. 13. The radiation pattern is extracted for operational frequencies of 5.5 GHz, 18 GHz, 28 GHz, 38 GHz, and 45 GHz, observed in the X–Z plane and X–Y plane, specifically in the Phi = 0° (H-plane) and theta = 90° (E-plane) orientations. The results are tabulated in Table 2. From this tabulated information, it becomes evident that both simulation and measured outcomes showcase a directive radiation pattern in the H-plane, with a close-to-omnidirectional behavior. Meanwhile, in the E-plane, the radiation pattern exhibits a bidirectional and stable nature.

**Table 2:** Far field characteristics of proposed UWB antenna at different frequencies in the XZ and XY planes (Simulated (solid line) – Measured (dash line).

*F(GHz)*	*Phi* = *0*	*3D Radiation*	*Phi* = *90*	*3D Radiation*
5				
16				
28				
38				
44				

In Table 2, The patterns of the proposed MIMO antenna which consists of a standard receiving horn antenna as reference and proposed work, which are both rotated 360°. The comparison between measurement and simulation results is satisfactory, with minor variations observed in both the H-plane and E-plane radiation patterns. These discrepancies can be attributed to factors such as environmental influences from nearby devices, radiation effects, and electromagnetic interference originating from the MIMO antenna elements.

To corroborate the accuracy of the simulated outcomes, a tangible version of the proposed design has been constructed. The constructed MIMO antenna, as illustrated in Fig. 9, accurately represents the fabricated model. Notably, the middle pin of the SMA connector is affixed at the precise center of the microstrip feedline.

**Fig. 14:**
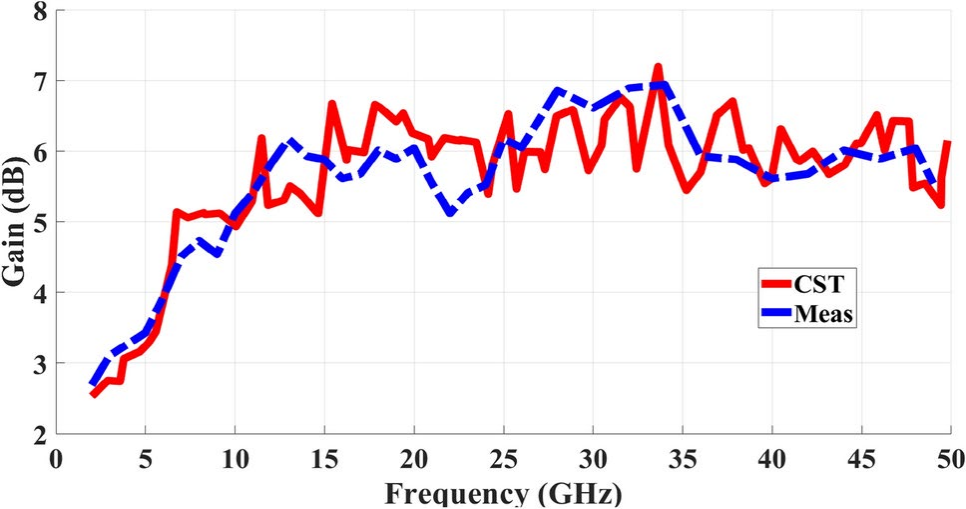
Gain Simulated and measured of the suggested MIMO antenna.

**Fig. 15:**
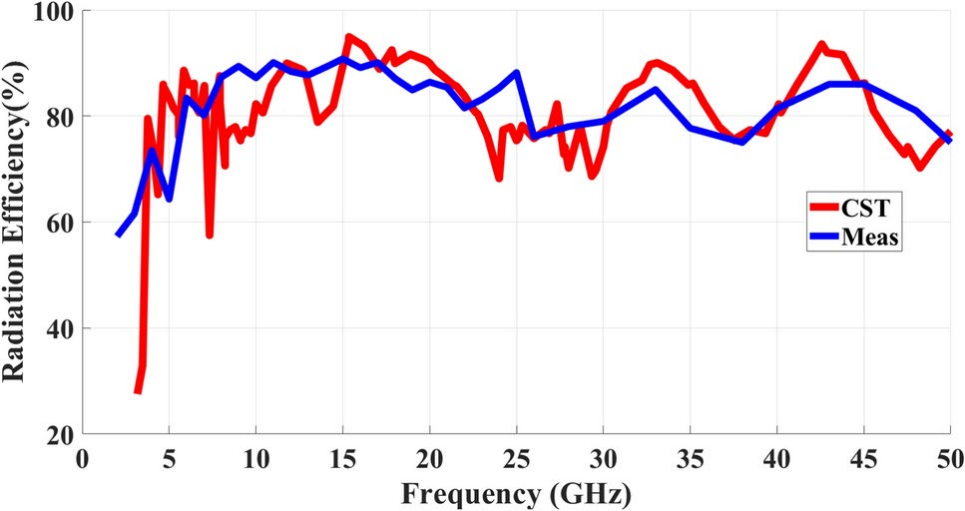
Radiation efficiency measurements and simulations for the suggested antenna.

The gain characteristics of the proposed MIMO antenna are depicted in Fig. 14. The antenna exhibits a moderate gain of more than 3.5 dBi across its operational bandwidth. Notably, at resonant frequencies, the gain attains peak values of approximately 6 dBi. In Fig. 15, a comparison is presented between the antenna’s radiation efficiency as measured and as simulated. Radiation efficiency gauges an antenna’s ability to convert supplied electrical power into electromagnetic radiation that propagates through free space. This metric reflects the ratio of the antenna’s output power to the total input power it receives. In essence, radiation efficiency quantifies how effectively an antenna transforms its input energy into usable radiation while minimizing energy loss as heat or stored within the antenna structure. This level of efficiency is attributed to the low loss tangent value of 0.0009 for the Rogers dielectric material utilized in the antenna.

### Measurement setup

This section aims to validate the simulation outcomes and draw a comparison with the results obtained through antenna simulation. To achieve this, the MIMO antenna was physically manufactured and subjected to experimental testing. The measurements were conducted using a vector network analyzer (VNA), specifically the R&S ZVA 67 as in Fig. 13. For the fabrication process, the main board was produced using photolithography technology on a Rogers 3003 substrate with parameters εr = 3 and tan δ = 0.02 as in Fig. 12. The substrate possessed a thickness of 1.575 mm. In accordance with the simulation results, the proposed design operates across a frequency spectrum spanning from 2.5 to 50 GHz. The performance observed between simulation and practical fabrication results demonstrated close alignment. Additionally, the MIMO antennas are connected to 50 SMA connectors located on the back surface of the printed circuit board.

### MIMO characteristics

In this section, we will delve into the fundamental performance parameters associated with a MIMO system. These characteristics hold paramount importance for a well-functioning MIMO setup. The key parameters under consideration are the Envelope Correlation Coefficient (ECC) and Diversity Gain (DG). The Envelope Correlation Coefficient (ECC) quantifies the extent to which the performance of one antenna remains independent of the performance of another antenna. Under ideal circumstances, the ECC value should be around 0.1. However, for practical applications, an ECC value of less than 0.5 is considered acceptable. The ECC for the proposed 2-port MIMO antenna arrangement, positioned side by side in opposing orientations, can be determined using the following formula^22^:

1 $$ECC_{ijFar - field} = \frac{{\iint {\left[ {E_{i} \left( {\theta ,\varphi } \right)*E_{j} \left( {\theta ,\varphi } \right)} \right]d\Omega^{2} }}}{{\iint {\left| {E_{i} \left( {\theta ,\varphi } \right)} \right|^{2} d\Omega \, \iint {\left| {E_{i} \left( {\theta ,\varphi } \right)} \right|^{2} d\Omega }}}}$$

**Fig. 16:**
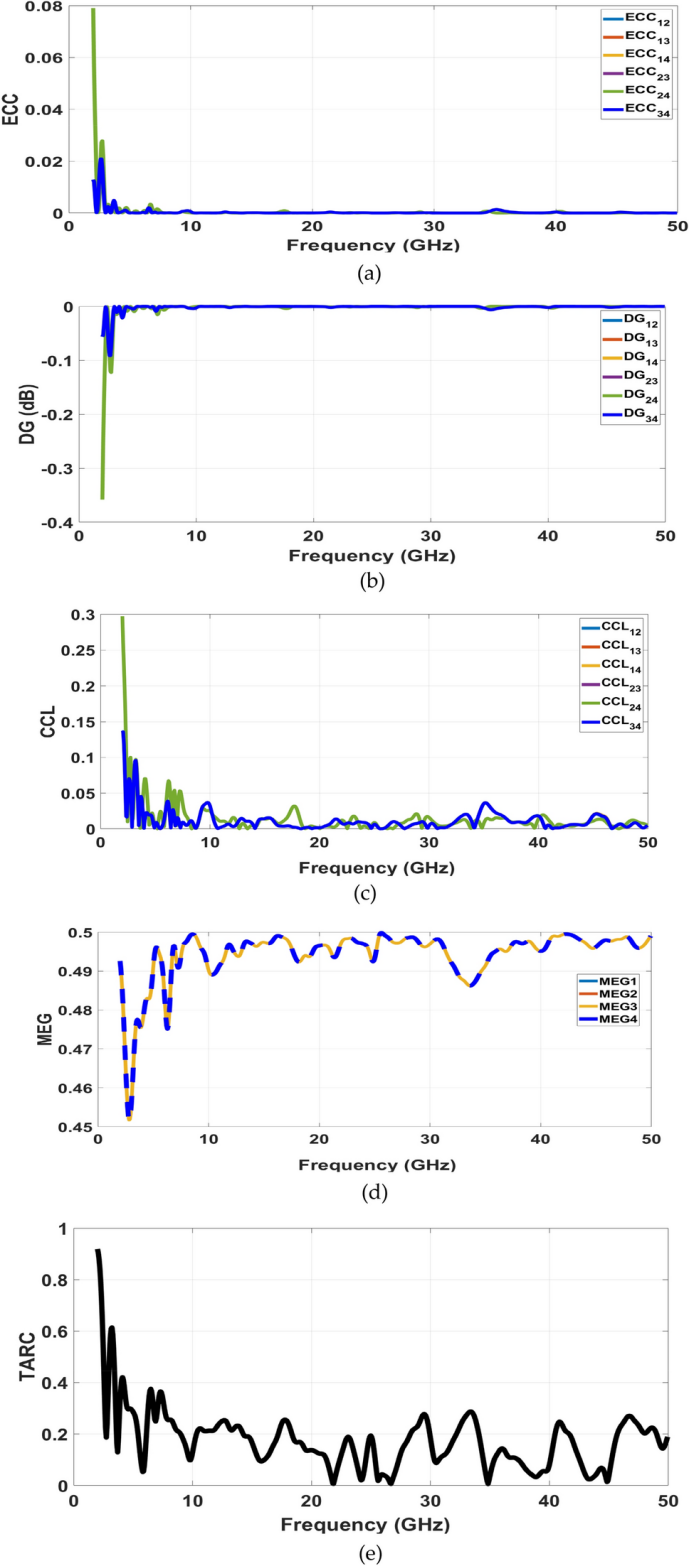
The proposed MIMO antenna performance parameters. (**a**) ECC (**b**) DG (**c**) CCL, (**d**) MEG, and (**e**) TARC.

Where $E_{i} \left( {\theta ,\varphi } \right)$ and $E_{j} \left( {\theta ,\varphi } \right)$ denote the complex 3D radiated electric field pattern of the ith and jth antennas, respectively, with i, j = 1, 2 for the proposed two-element MIMO antenna. Figure 16(a) Illustrated, the ECC values for our proposed configuration have been computed using both simulation data and measurements. Notably, the measured ECC is notably below 0.013, which is attributed to the considerable isolation between the radiating elements within the MIMO setup. Diversity Gain (DG) represents the reduction in transmission power that occurs when diversity techniques are implemented in a MIMO antenna system. This parameter is pivotal for evaluating the performance of MIMO antenna systems. In terms of compactness, multiband operation, isolation, maximum ECC, and gain diversity, our MIMO antenna clearly outperforms the suggested four-port MIMO antenna. The DG value can be computed using the formula from^22^:

2 $$\text{DG}=10\sqrt{1-{(\text{ECC})}^{2}}$$

As demonstrated in Fig. 16(b), all the antenna ports exhibit a diversity gain surpassing 9.995 dB, a value very close to the ideal 10 dB.

As in Fig. 16(c), the channel Capacity Loss (CCL) is a crucial metric in MIMO systems. It represents the potential reduction in channel capacity caused by the correlation between MIMO links. One approach to calculating the CCL is outlined in Eq. (3), as reported in^12^.

3 $$\text{CCL}= -{\text{log}}_{2}\left[\text{det}(\text{a})\right]$$

where $$\text{a}= \left[\begin{array}{cc}{\upsigma }_{11}& {\upsigma }_{12}\\ {\upsigma }_{21}& {\upsigma }_{22}\end{array}\right]$$ ,

$$\sigma_{i} i = 1 - (|S_{i} i|^{2} - |S_{i} j|^{2} ),and,$$

$$\sigma_{i} j = - \left( {S_{i} i^{*} S_{i} j + S_{j} iS_{j} j^{*} } \right)$$

The MEG and TARC values are extracted and computed using the equations from^27,28^, and the results are shown in the simulated data presented in Fig. 16(d) and Fig. 16(e), respectively.

The MEG for the antenna, shown in Fig. 15(d), reaches a value of − 3 dB at all four ports within the operating frequency range. A similar outcome is shown in Fig. 16(e) for the bent MIMO antenna. The CCL of the proposed MIMO antenna falls within the range of 0 to 0.2 bits/sec/Hz across the operating frequency band.

### Simulated time domain analysis

The performance of the proposed antenna is assessed using time-domain analysis. In UWB technology, transmitting a narrowband pulse leads to a wideband response in the frequency domain. As a result, key time-domain parameters like the transmission coefficient S21, the phase of S21, and group delay are essential for evaluating the antenna’s effectiveness for super UWB applications.

**Fig. 17:**
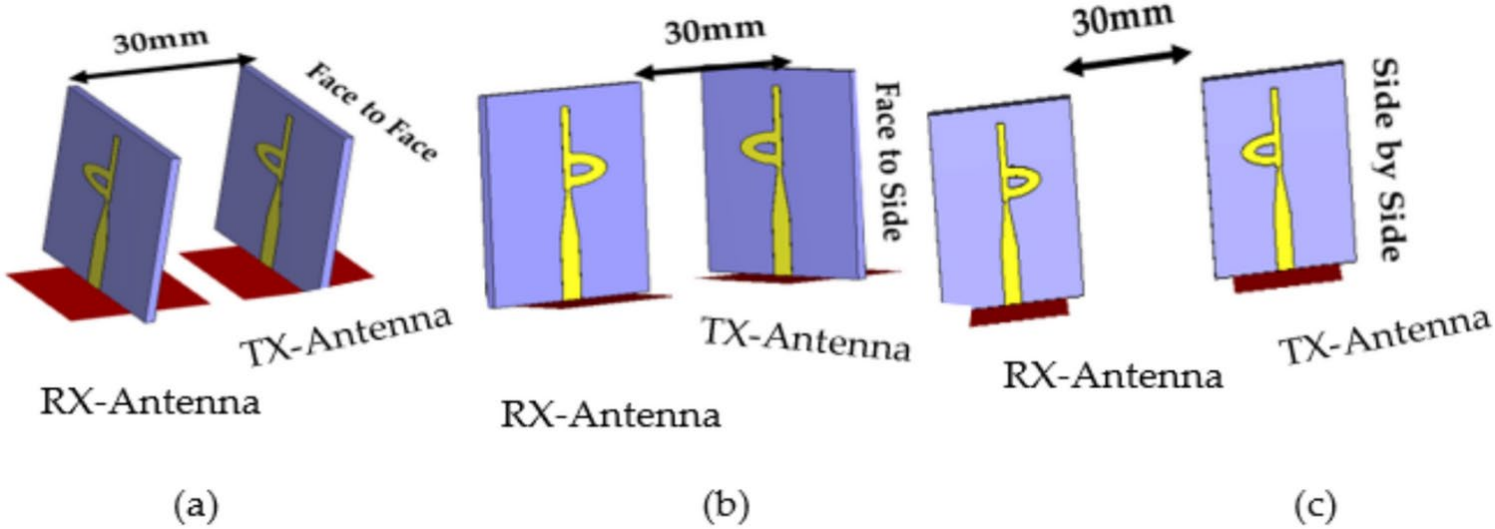
Simulated time-domain analysis setup at different layout, (**a**) Face to Face, (**b**) Face to Side, and (c) Side to Side.

Figure 17 illustrates the simulated time-domain setup for three distinct configurations: side-by-side, face-to-side, and face-to-face. In each arrangement, two identical antennas are positioned 30 mm apart, which corresponds to 2.45 times λ0 at 3.5 GHz. One antenna operates as the transmitter (Tx), while the other functions as the receiver (Rx).

**Fig. 18:**
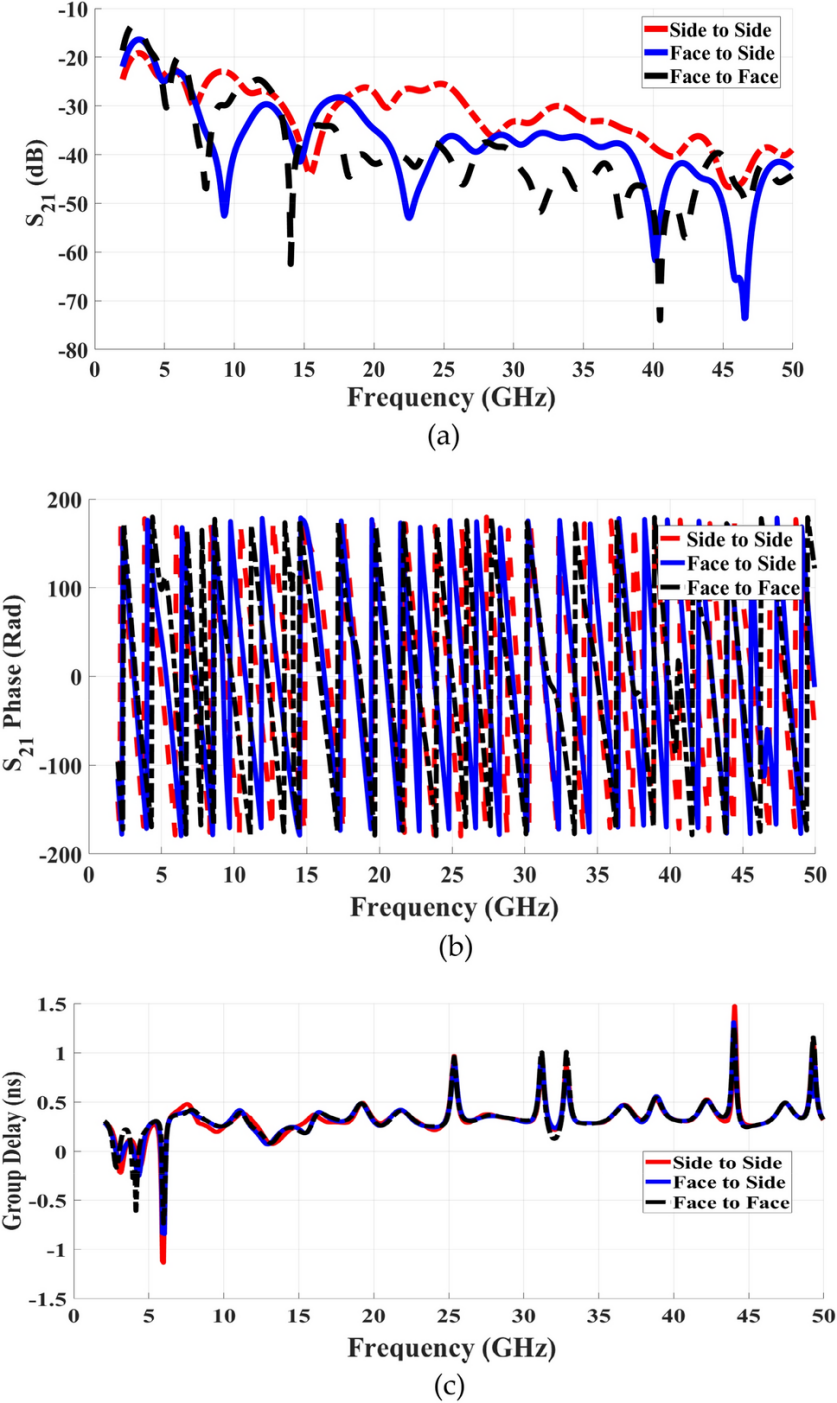
Simulated results at the different layout (**a**) S21 Magnitude., (**b**) S21 Phase, and (**c**) Group Delay.

Figure 18 presents the magnitudes of S21, and the S21 phase. As shown in Fig. 17, all three antenna configurations exhibit S21 values below − 25 dB across the entire frequency range, maintaining stable values range between 5 to 30 GHz. However, in the higher frequency range from 25 to 50 GHz, S21 values drop further to below − 30 dB, and -40 dB particularly for the face-to-face and face-to-side configurations. Additionally, the S21 phase is shown in Fig. 18b, and given to better analyze the linearity characteristics over the wideband operation.

The antenna operates linearly within the frequency spectrum at various configurations, as can be seen from the phase curves. Figure 18 c shows the group delay results for further confirmation. Particularly at the two face-to-face and face-to-side configurations, it is observed that the group delay has a consistent value of around 1.31 ns within the working frequency band with a variance of about 0.1 ns at the end of the band.

Table 3 provides a comprehensive overview of the comparison between the proposed MIMO antenna and recently published mm-wave MIMO antennas. The proposed super UWB MIMO antenna system exhibits remarkable performance across various metrics, boasting the best isolation, gain, impedance bandwidth, and ECC values. It does, however, possess slightly lower ECC values when compared to the antenna with the most favorable performance metrics. This indicates that our suggested antenna design outperforms the other discussed antennas in terms of MIMO performance. Furthermore, a comparison between the proposed MIMO antenna and other reference MIMO antennas functioning within the sub-6 GHz range is detailed in Table 3. shows how the proposed study compares to other earlier works. Because of its straightforward design, good isolation, and high gain, the proposed work is likely to be used in super UWB MIMO systems. Collectively, these outcomes substantiate the notion that the proposed MIMO antenna system holds significant promise as a suitable contender for the future 5G mobile terminal industry.

**Table 3:** Comparison between suggested and literature works.

Ref [Year]	Size (mm^3^)	Size (λ_g_)^2^	Substrate	Fre BW (GHz)	Isolation (dB)	Gain (dBi) Avg	ECC/DG (dB)	Appli
^12^ 2020	80 $\times$ 80 $\times 1.6$	1.24 × 1.24	FR4$( \in_{r} = {4}.{4})$	22–27	-15	5.8	0.01/9.96	WiMax /radar applications
^15^ 2020	150 $\times 100\times 0.$ 8	0.76 × 0.68	RT4350B$( \in_{r} = {3}.{66})$	2–5	-17	6.1	0.32/9.7	Mobile Comm
^19^ 2020	80 $\times 80\times$ 1.6	0.87 × 0.76	RT5880$( \in_{r} = {2}.{2})$	20–40	-20	8.5	0.0019/9.8	5G
^5^ 2021	$93\times 93\times 1.6$	1.22 × 1.22	FR4$( \in_{r} = {4}.{4})$	26–31	-18	5.3	0.012/9.19	5G NR networks
^23^ 2023	36 $\times 27\times 1.6$	0.98 × 0.73	RT4003 $\left( { \in_{r} = {3}} \right)$	7.9–9.5	-18	3.55	0.01/9.25	X-band
^24^ 2021	$116\times 84\times 1.6$	0.72 × 0.84	FR-35$( \in_{r} = {3}.{5})$	2.5–20	-17.5	5.4	0.044/8.4	WLAN- Satellite systems
^25^ 2023	90 $\times 90\times$ 1.6	0.87 × 0.76	RT5880$( \in_{r} = {2}.{2})$	5–9	-17	5.65	0.09/9.3	sub-6 ghz v2x comm
^26^ 2021	30 × 40 × 1.6	0.6 × 0.48	FR4 $( \in_{r} = {4}.{4})$	3.34–9.2	-20	3.5	0.05/9.98	sub-6 GHz and WLAN bands
^27^ 2022	55 × 55 × 1.6	0.612 × 0.612	FR4 $( \in_{r} = {4}.{4})$	3.34–9.2	-20	4.3	0.017/8.9	sub-6 GHz 5G and X band
^28^ 2022	24 × 24 × 1.6	3. 12 × 3.12	RT5880$( \in_{r} = {2}.{2})$	24.8–44.45	-20	8.6	0.008/8.5	sub-mm5G New Radio (NR)
^29^ 2022	15 × 15 × 1.6	2.01 × 1.95	RT5880 $( \in_{r} = {2}.{2})$	26–40	-20	7	0.02/9	mm-wave 5G
^30^ 2023	36 $\times 27\times 1.6$	0.98 × 0.73	RT4003$( \in_{r} = {3})$	7.8–6.5	-16.5	3.55	0.01/8.5	X-band
^31^ 2023	12 × 10 × 0.8	2.24 × 2.24	RT5880$( \in_{r} = {2}.{2})$	27.12–31.34; 37.21–38.81	-15	5.7	0.04/9	mm-wave 5G
^32^ 2024	160 $\times 70*0.214$	0.64 × 0.70	RT4003$( \in_{r} = {3})$	4–5.8	-12	5.3	0.05/9.7	UWB
^33^ 2024	82 $\times 82*1.6$	0.64 × 0.70	RT5880$( \in_{r} = {2}.{2})$	3–40	-15	12.5	0.09/9.8	Indoor Comm
^34^ 2020	190 $\times 80*0.8$	2.2 × 1.86	FR4$( \in_{r} = {4}.{4})$	3–35	-20	6.75	0.09/9	microwave applications
^35^ 2021	116.5 $\times 84.5*1.575$	1.82 × 1.32	FR 3$( \in_{r} = {3}.{5})$	3.5–30	-15	5.2	0.015/–	sub-6 GHz and 5G
**This Work**	70 × 70 × 1.575	0.62 × 0.62	RT4003$( \in_{r} = {3})$	2.5–50	-25	8.5	0.013/9.9	IOT, Sat. Comm., 5G and Beyond Applications

## Conclusion

The design of the antenna employs a radiating patch in a d-shaped configuration and a partial ground plane formed as a semicircle. The antenna’s operational frequency range spans from 2.5 to 50 GHz, Additionally, MIMO antennas can increase its data rate. As a result, the suggested antenna is appropriate for mmWave applications that span several wireless bands, including the 5G, sub 6G, and satellite bands that are supported by IoT applications. exhibiting notable isolation (> 25 dB). The systematic approach towards crafting an antenna system for 5G wireless communication is underscored by the conducted parametric analysis and surface current distribution assessment, which aimed to optimize parameters and understand coupling between elements. Thorough investigations into key design parameters using the proposed methodologies, alongside other attributes, provide a comprehensive understanding of the MIMO antenna design. Careful analysis and optimum dimensions are determined through simulation using the 3D EM simulator CST. There exists a strong correlation between experimental and simulated outcomes. The design is implemented on a 70 × 70mm^2^ Roger 3003 substrate possessing a dielectric constant of 3, thickness of 1.6 mm, and a tangent loss of 0.002. To mitigate mutual coupling, orthogonal orientation of resonators is utilized. Performance metrics such as radiation pattern, isolation, ECC, and DG are compared against standard values as previously described. In summary, the proposed antenna offers distinctive advantages and potential applications, marking a substantial stride forward in antenna design. The antenna boasts a high gain of up to 9 dB and exceptional efficiency reaching up to 87%, radiating bidirectionally in both the E- and H-planes.

## Data Availability

Data Availability: The datasets used and analyzed during the current study are available from the corresponding author on reasonable request.
